# Consumption of Sprouts and Perceptions of Their Health Properties in a Region of Northwestern Mexico

**DOI:** 10.3390/foods10123098

**Published:** 2021-12-14

**Authors:** Alan Amado Ruiz Hernández, Fernando Ayala Zavala, Ofelia Rouzaud Sández, Juana Frias, Humberto Astiazarán-García, Rosario Maribel Robles Sánchez

**Affiliations:** 1 Departamento de Investigación y Posgrado en Alimentos, Universidad de Sonora, Blvd. Luis Encinas y Rosales, Colonia Centro, Hermosillo 83000, Sonora, Mexico; alanamado12@hotmail.com (A.A.R.H.); ofelia.rouzaud@unison.mx (O.R.S.); 2 Centro de Investigación en Alimentación y Desarrollo, A.C., Carretera Gustavo Enrique Astiazarán Rosas, No. 46, Colonia La Victoria, Hermosillo 83304, Sonora, Mexico; jayala@ciad.mx (F.A.Z.); hastiazaran@ciad.mx (H.A.-G.); 3 Instituto de Ciencia y Tecnología de Alimentos y Nutrición, Calle José Antonio Novais, 10, 28040 Madrid, Spain; frias@ictan.csic.es

**Keywords:** survey, sociodemographic, schooling, income, people

## Abstract

There is a lack of information about consumer understanding of functional foods. Sprouts provide beneficial compounds that can help counteract chronic noncommunicable diseases. The population of a region in Northwestern Mexico has a high prevalence of chronic degenerative disease, and there is a need to promote strategies to increase the consumption of foods that provide health benefits, including sprouts. However, there is a lack of information regarding the sale, consumption and perception of sprouts’ healthy properties. A computer-assisted web-based survey (CAWI) was developed and distributed through social media to understand consumer knowledge of these foods’ health effects and their consumption. The survey of people with diverse sociodemographic profiles indicated a 1–3 times per week consumption and they knew the health benefits of consuming sprouts. A total of 82% of respondents were conscious that sprout consumption could prevent chronic diseases, which may be related to education level (χ^2^: 0.001, *p* < 0.05). In order to expand on our findings, it is important to investigate the communication strategies used by sprout manufacturers, dieticians, nutritionists and health professionals about the health benefits of sprout products to promote their consumption.

## 1. Introduction

In recent years, the high prevalence of chronic degenerative diseases and changes in lifestyles have led to new food consumption trends. Consumers are turning towards diets rich in fruits and vegetables characterised by a high content of bioactive compounds. Sprouted seeds are ready-to-eat vegetables that have been harvested at very early stages of growth [1]. As sprouts are consumed at the beginning of the growing phase, their nutrient concentration remains very high [2]. In addition to being a rich source of nutritional compounds, sprouts contain as many phytochemicals (sulphoraphane, sulphoraphene, isothiocyanates, glucosinolates, enzymes, antioxidants and vitamins) as an entire plant [3,4]. Sprouted seeds have long been consumed in oriental countries and form an important part of their culinary history. Given their rich flavour, smell and bioactive compounds, these products can be used to prepare salads or other high-quality foods, and positive perceptions of these products have influenced the development of foods such as breakfast cereals, snacks and baked goods [5]. However, although there are new options for the consumption of sprouts and a large proportion of the population prefers healthy foods and is inclined to consume them, several studies have reported that the selection and perception of healthy foods could be related to several sociodemographic characteristics of the population [6].

Several countries have developed study models to evaluate consumer health perceptions concerning functional foods [7], but very few studies have focused on the consumption of germinated seeds. The manufacturers of sprouted products continue developing new products based on sprouts, and dietitians and nutritionists recommend them as part of a healthy diet, but the level of preference, consumption and health perception that consumers have is still unknown. Mexico has a high prevalence of obesity and overweight (71% and 37% in adults and childhood, respectively). As obesity is a risk factor for the development of several chronic diseases [8], it is urgent to design public health strategies that promote healthy eating to prevent and treat these diseases.

According to the findings reported by previous studies about the influence of sociodemographic variables on factors that determine consumption, the perception of health and knowledge of sprouted grains and that these relationships may behave differently depending on the customs, beliefs and culture of each country, it is possible to formulate the following hypothesis: the sociodemographic profile of a population in northwestern Mexico influences the perception of health, knowledge and frequency of consumption of germinated seeds.

The main goal of this study was to examine the influence of sociodemographic variables such as gender, age, education level, occupation and monthly income on frequency of consumption of germinated seeds and knowledge about the health benefits that these foods provide, through the use of surveys aimed at the adult population in a semi-industrial region with a prevalence of obesity above the national average.

## 2. Materials and Methods

Due to the COVID-19 pandemic conditions, a computer-assisted web interview (CAWI) survey was applied to the population of Hermosillo, Sonora, Mexico. The Google Forms^®^ platform was used to develop the survey, and social networks (Facebook and WhatsApp) were used for its distribution.

### 2.1. Survey Development

The survey and questions were based on the recommendations of Anguita et al. [9] with slight modifications, including closed questions that reflect the participant’s opinion, multiple-choice questions with a range of answers, and estimation questions. In addition, participants were asked to evaluate sprout attributes in terms of their preference when purchasing on a 5-point Likert scale: 1 = I like it very much, 2 = I like it, 3 = I am indifferent, 4 = I dislike it, and 5 = I dislike it very much. To learn about the sprouts offered for sale, a local commercial store was visited (Casa Ley^®^), as well as the online store of the producer San Francisco^®^. A detailed description of the questionnaire is included in Table S1.

### 2.2. Experimental Design and Statistical Analysis

**Figure 1:**
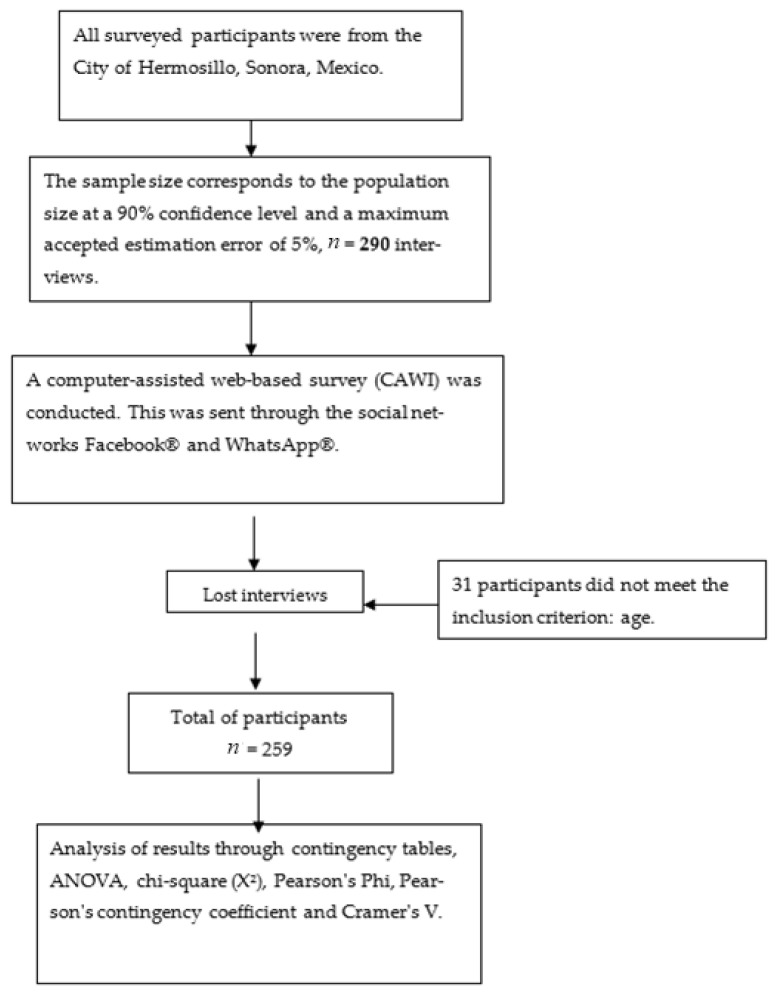
General scheme of study design.

For the sample size, the number of inhabitants of Hermosillo, Sonora México was considered (*N* = 936,263; INEGI, 2020), the confidence level was set at 90%, and the maximum accepted estimation error was 5% [10]. The sample size was *n* = 290. Simple random sampling with a cross-sectional dimension and descriptive research method were used. For the study of the variables, contingency tables were used, where the association between variables was tested using the chi-square statistical test (χ^2^ at level *p* < 0.05). Pearson’s phi, Pearson’s contingency coefficient and Cramer’s V tests [11] were used to determine the strength of the association between variables. IBM SPSS 25^®^ statistical software was used to study the results and develop graphs (Figure 1).

## 3. Results and Discussion

### 3.1. Socioeconomic Variables

After age exclusion, the final sample (*N* = 259) consisted of 178 (68.7%) women and 81 (31.2%) men. The largest age group for women was 18–28 years (24.7%), whereas for men, 29–39 years was the largest group (11.6%). In terms of education, 36.7% of women and 16.2% of men had a university education, and 26.6% of women and 12.7% of men had postgraduate education. About half of the participants had a personal monthly income between MXN $5000 and $25,000. Table 1 describes these sociodemographic characteristics in detail.

### 3.2. Perception of Sprout Sales and Consumption

Participants were given a list of sprouts to know which were available on the market. Among them were soybeans, radish, alfalfa and broccoli. Alfalfa, mung beans, soybeans, lentils and broccoli were the most easily identified sprouts. According to the Oregon Public Health Division [12], these data are consistent with the most widely produced sprouts, in which radish, wheatgrass, broccoli, mung bean, red clover and alfalfa are included. Other species such as lentils, soybeans, barley, corn, carrots, peas, turnips, sesame, sunflower, quinoa and almonds are also used to produce sprouts [13]. Within this same list, they were asked to identify which they consumed. The results showed that 87% of the participants consumed more than one sprout, 8.1% responded that they had only consumed one of the sprouts on the list and 4.2% had not consumed any of the sprouts from the given list.

According to Statista reports [14,15], sprouts are among the healthiest foods consumed by the Mexican population: of 7000 interviewed, 30.1% ate sprouts at least once a week, 45.5% consumed them three or four times a week, 15.8% consumed them five or six times a week and 6.6% ate them daily. In the same survey, other healthy products consumed were salads with chicken breast, steamed vegetables and vegetable soup.

### 3.3. Knowledge, Consumption and Health Perception of Sprouts

The sensory attributes of sprouts are criteria that significantly influence the purchase decision. To understand the preferences of respondents in relation to sensory attributes of the germinated seeds, a five-point Likert scale was applied to evaluate perceptions of flavour, texture, odour, colour and consistency. All participants surveyed rated the attributes of the sprouts with values between 1 (I like it very much) and 3 (I am indifferent), which implies that there is a positive perception regarding the sensory attributes of sprouts (Table 2).

When values were grouped by age range and gender, it was observed that participants in the highest age range gave the strongest preferences to the attributes. This was significant for women (*p* < 0.05) but not for men. This result could be related to the fact that women are more commonly responsible for providing and preparing food, which may provide them with greater knowledge of sprouted products. This is consistent with Sääksjärvi et al. [16], who demonstrated that females were more likely to be knowledgeable than males about functional foods.

**Figure 2:**
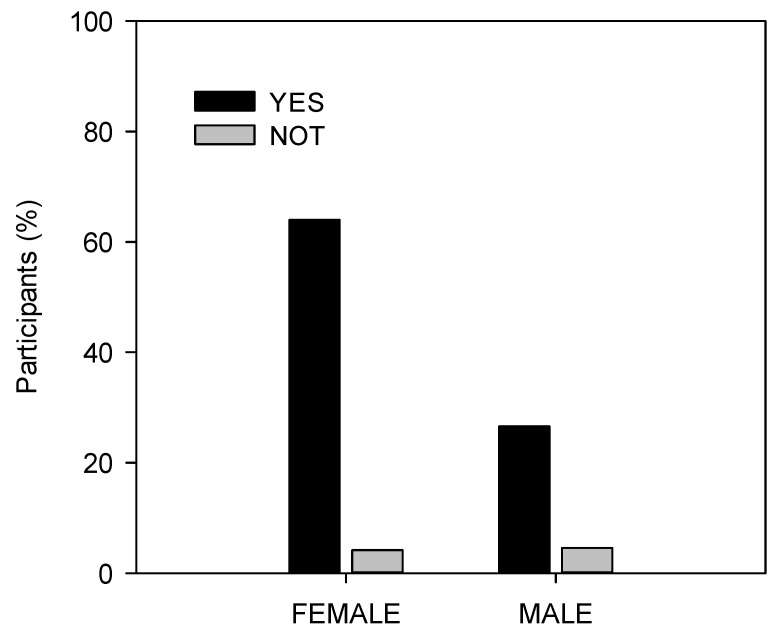
Percentage of participants by gender who would encourage consumption of sprouts to prevent disease.

As previously mentioned, the associations between sociodemographic variables and knowledge, consumption and health perception of sprouts were evaluated. A significant association was found (chi-square 0.024, *p* < 0.05) between gender and the decision to consume or increase consumption of sprouts, knowing the health benefits attributed to these products. Although the strength of the association was weak (Phi: −0.14; Cramer’s V: 0.14; contingency coefficient: 0.13), it was observed that women answered affirmatively more often than men (Figure 2). This gender disparity could be related to the higher rate of life-threatening diseases prevalent in women (36%) than in men (10%) (chi-square 0.002, *p* < 0.05).

**Figure 3:**
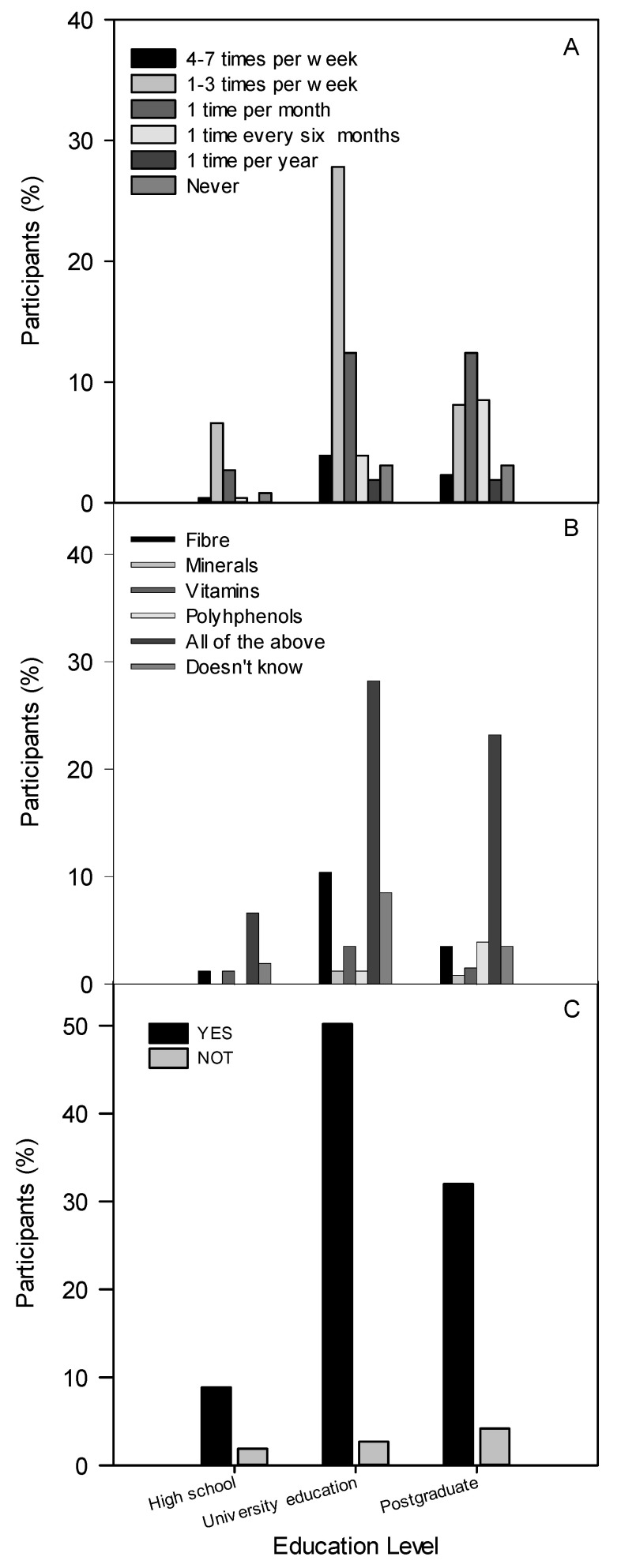
Percentage of participants by education level who responded to questions: (**A**) How often do you consume sprouts? (**B**) To what would you attribute the beneficial effect of sprouts? (**C**) Would you consume or increase the consumption of sprouts to prevent these diseases?

The relationship between education and frequency of sprout consumption was significant (chi-square < 0.001, *p* < 0.05), with a strong association according to Phi: 0.357, Cramer’s V: 0.253 and contingency coefficient: 0.336. This means that a high percentage of respondents with higher academic levels indicated a higher frequency of sprout consumption at 1–3 times a week and once a month. According to these results, it is also possible to highlight that all respondents were not frequent consumers of sprouted products (Figure 3A). This is consistent with a cross-sectional study conducted in France, where favourable attitudes towards healthy food consumption, diet quality and educational level were associated with a higher level of good health [17].

To identify whether participants were aware of the beneficial effects attributed to compounds present in sprouts, they were asked several questions. The results were as follows: 57.9% indicated that these effects were due to all suggested options, 15% said fibre, 13.9% were completely unaware of them, 5% responded with polyphenols, 6.2% attributed them to vitamins and 1.9% said minerals. Education level also showed a significant association (chi-square 0.04, *p* < 0.05) with participants’ knowledge in relation to the beneficial compounds. Approximately 30% and 25% of the responses given by participants with university and postgraduate studies, respectively, identified fibre, minerals, vitamins and polyphenols as components that contributed to the beneficial effects of sprouts (Figure 3B).

Another significant association was found between education level and a person’s decision to consume or increase consumption of sprouts, knowing the health benefits attributed to this product (chi-square 0.047, *p* < 0.05). Participants with higher education levels gave affirmative responses more often to this question (Figure 3C).

**Figure 4:**
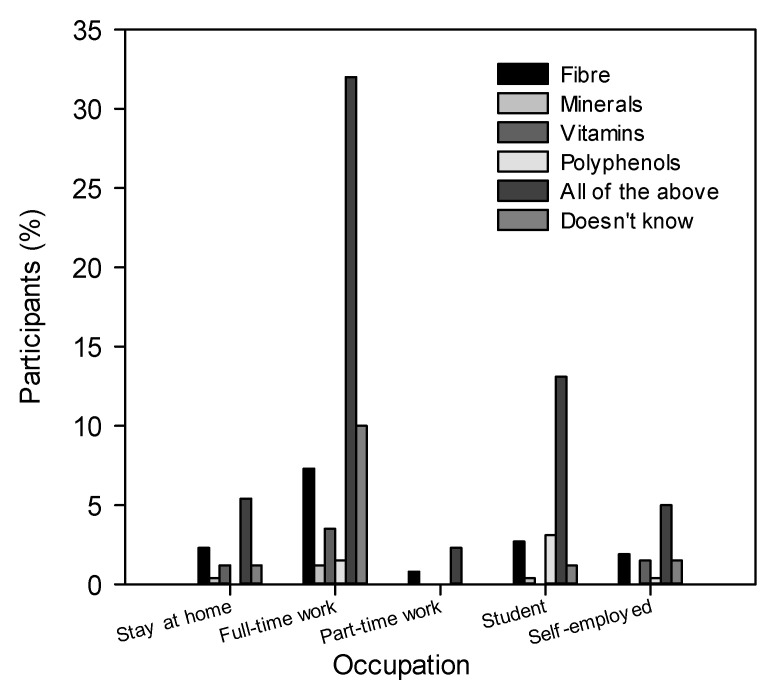
Percentage of participants by occupation who responded to question: To what would you attribute the beneficial effect of sprouts?

**Figure 5:**
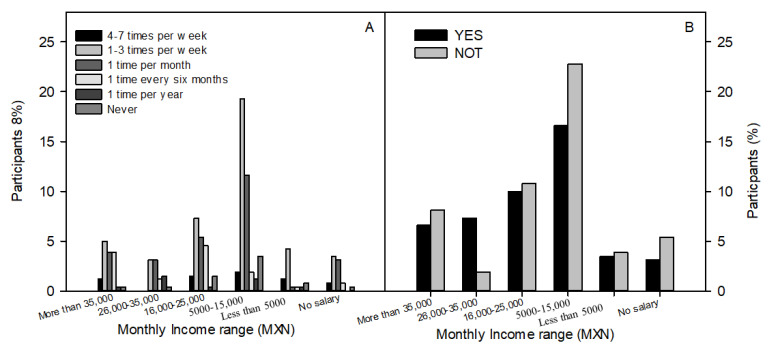
Percentage of participants by monthly income range who responded to questions: (**A**) How often do you consume sprouts? (**B**) Regular consumption of sprouts is linked to a lower likelihood of developing diseases such as diabetes, hypertension and cancer. Did you know this?

A significant association was observed (chi-square 0.039, *p* < 0.05) between the occupation of the participants and knowledge of the components contributing to the beneficial effects of sprouts. It was observed that most of the participants demonstrated to have knowledge of all the compounds suggested on the list and to which health effects are attributed. The highest percentages corresponded to those participants with full-time work (Figure 4). This is related to the significant association (chi-square 0.014, *p* < 0.05) between monthly income reported by respondents and frequency of sprout consumption. It was observed that participants consumed sprouts more frequently (1–3 times a week; once a month) as their salary decreased, but this behaviour changed to a decreased frequency of sprouts when the income was less than MXN $5000 or they did not receive a salary (Figure 5A). In this regard, it should be noted that of the participants surveyed, 20% responded to having full-time work with a monthly income of MXN $5000–$15,000. This coincides with the highest frequency of sprout consumption (20% and 12% for consumption frequencies of 1–3 times a week and once a month, respectively).

In contrast, a study that evaluated knowledge of the benefits of legumes and their consumption showed that despite presenting theoretical knowledge, the choice to use them in food preparation was low [18], which was attributed to the difficulty of preparation and aversion to these foods.

The knowledge of sprouts being preventative for several diseases was evaluated with a yes/no question. This was significantly associated (chi-square 0.034, *p* < 0.05) with the monthly income range of participants. An increase was observed in the percentage of participants who did not know the health benefits of sprouts as monthly income decreased, and the highest levels of ignorance were observed in participants with a monthly income of MXN $5000–$15,000 (Figure 5B). Participants in this income range and who mostly had full-time work frequently consumed sprouts but were unaware of the health benefits. This level of income is considered medium-range, according to Rankia [19]. In comparison, a study by French et al. [20] indicated that the lower the income, the lower the quality of diet consumed.

Although education and income levels may influence the consumption of healthy foods such as sprouts, the eating habits of school-aged youth may be affected by food availability, school schedules and peer pressure [21]. This effect could influence the interest of young people in healthy eating, and although they know about good eating and healthy habits, they may not carry them out [22]. Though information on people’s knowledge about the qualities of sprouts has been lacking so far, it is known that people come to establish healthy habits through their personal, cultural, social and environmental experiences [23], thus it is expected that these findings are similar for sprouts.

Results indicated that the studied group was conscious of the health benefits of sprouts, mainly by relating them to vegetables. However, even when the consumption of vegetables (including sprouts) is considerable in the Mexican population, the large portion size of consumed food, and the lack of physical activity, have increased the prevalence of overweight and obesity in men (+5.1%) and women (+4.1%) over 20 years old from 2012–2018 [24]. Even when there is the purchase intention, there are few habitually con-sumed foods prepared with sprouts; therefore, it is necessary to develop new products with acceptable organoleptic, nutritional, and functional aspects.

For the entire sampled population, most of the variables associated with knowledge, perception of health and consumption of sprouts were not strongly influenced by gender; however, when analysing the survey data separately in groups of female and male, a greater number of significant associations were observed in the group of women, who showed that a medium monthly income and high educational level corresponded to a higher frequency in the consumption and perception of the health of germinated seeds (Table 3). This is in line with other studies where women are more active in the promotion, provision, consumption and knowledge of healthy foods, among which sprouted seeds could be included [17,25].

## 4. Conclusions

Sociodemographic variables such as education level, occupation and monthly income showed significant influence on knowledge, health perceptions and consumption of sprouted seeds. However, a proportion of population with high education level, medium monthly income and with full time work mentioned having no knowledge of the health benefits of sprouts. Knowing that half of the participants regularly consumed sprouts or had the intention to consume them for their beneficial effects and high sensory acceptability, it is possible to work on the development of new foods and production of sprouts from non-traditional seeds. Further research on their beneficial effects can inform the development of programmes and public policies that reinforce the benefits of healthy food products, particularly for people with chronic noncommunicable diseases. It is important to highlight that the results of our research should be considered ex-ploratory and preliminary, and future research may study other factors and territories.

## Figures and Tables

**Table 1:** Sociodemographic characteristics of study population (*N* = 259) ^1^.

Characteristics	Female 178 (68.7)	Male 81 (31.2)
Age		
18–28	64 (24.7)	21 (8.1)
29–39	48 (18.5)	30 (11.6)
40–50	30 (11.6)	10 (3.9)
51–60	36 (13.9)	20 (7.7)
Education level		
High school	22 (8.5)	6 (2.3)
University education	45 (36.7)	42 (16.2)
Postgraduate	61 (26.6)	33 (12.7)
Occupation		
Stay at home	27 (10.4)	0 (0.0)
Full-time work	36 (13.9)	17 6.6)
Part-time work	8 (3.1)	0 (0.0)
Student	92 (35.5)	52 (20.1)
Self-employment	15 (5.8)	12 (4.6)
Monthly income (MXN) range		
>$35,000	19 (7.3)	3 (1.2)
$26,000–$35,000	18 (6.9)	1 (0.4)
$16,000–$25,000	72 (27.8)	30 (11.6)
$5000–$15,000	36 (13.9)	18 (6.9)
Less than $5000	16 (6.2)	8 (3.1)
No salary	17 (6.6)	21 (8.1)

^1^ The values correspond to the frequency of responses for each variable. Values in parentheses correspond to the percentages on a basis of 259 participants (100%).

**Table 2:** Rating of sensory attributes of sprouts in terms of consumer preferences at the time of purchase ^1^.

	Flavour	Texture	Odour	Colour	Consistency
Female Age range					
18–28	2.76 ± 0.16 ^a^	2.76 ± 0.14 ^a^	3.00 ± 0.13 ^a^	2.79 ± 0.13 ^a^	2.84 ± 0.14 ^a^
29–39	2.47 ± 0.17 ^ab^	2.52 ± 0.16 ^ab^	2.85 ± 0.15 ^ab^	2.60 ± 0.15 ^ab^	2.52 ± 0.15 ^ab^
40–50	2.46 ± 0.18 ^ab^	2.33 ± 0.19 ^ab^	2.68 ± 0.15 ^ab^	2.43 ± 0.18 ^ab^	2.36 ± 0.18 ^ab^
51–60	2.08 ± 0.11 ^b^	2.13 ± 0.13 ^a^	2.33 ± 0.14 ^b^	2.19 ± 0.13 ^b^	2.25 ± 0.15 ^b^
Male Age range					
18–28	2.65 ± 0.22 ^a^	2.60 ± 0.23 ^a^	2.95 ± 0.18 ^a^	2.66 ± 0.22 ^a^	2.60 ± 0.20 ^a^
29–39	2.50 ± 0.17 ^a^	2.57 ± 0.17 ^a^	2.76 ± 0.14 ^a^	2.60 ± 0.18 ^a^	2.50 ± 0.11 ^a^
40–50	2.46 ± 0.22 ^a^	2.50 ± 0.30 ^a^	2.73 ± 0.26 ^a^	2.56 ± 0.26 ^a^	2.47 ± 0.26 ^a^
51–60	2.30 ± 0.25 ^a^	2.30 ± 0.19 ^a^	2.50 ± 0.21 ^a^	2.50 ± 0.24 ^a^	2.33 ± 0.25 ^a^

^1^ The values correspond to the mean ± standard error of the grades given by the surveyed participants for each of the sensory attributes of the sprouts. Different letters in each column between genders are statistically different at *p* < 0.05.

**Table 3:** Sociodemographic variables associated with consumption, knowledge and health perception of seed sprouts according to grouped data by gender survey ^1^.

Associated Variables	Chi-Square Statistical Test (χ2)	Pearson’s Phi	Cramer’s V Tests	Pearson’s Contingency Coefficient
**Female *(n* = 178)**				
Education level (Q3) * How often do you consume sprouts? (Q11) *	0.003	0.390	0.276	0.363
Education level (Q3) * Would you consume or increase your consumption of sprouts to prevent diseases? (Q14) *	0.003	0.258	0.258	0.250
Education level (Q3) * To what would you attribute the beneficial effect of sprouts? (Q12) *	0.013	0.356	0.251	0.335
Occupation (Q4) * How often do you consume sprouts? ((Q11) *	0.035	0.430	0.215	0.395
Monthly Income (Q5) * How often do you consume sprouts? (Q11) *	0.011	0.498	0.223	0.446
Monthly income (Q5) * Would you consume or increase your consumption of sprouts to prevent diseases? (Q14) *	0.031	0.262	0.262	0.254
Monthly income (Q5) * To what would you attribute the beneficial effect of sprouts? (Q3) *	0.042	0.465	0.208	0.425
**Male (*n* = 81)**				
Education level (Q3) * How often do you consume sprouts? (Q11) *	0.008	0.542	0.383	0.477
Education level (Q3) * Would you consume or increase your consumption of sprouts to prevent these diseases? (Q14) *	0.006	0.357	0.357	0.336
Physical activity (Q6) * Would you consume or increase your consumption of sprouts to prevent diseases? (Q14) *	0.028	0.334	0.334	0.317

^1^ Significant association between variables (χ2: *p* < 0.05); * (Q number) indicates the number of the question asked to the participants according to the questionnaire shown in Table S1 (Supplementary material).

## Data Availability

Not applicable.

## Supplementary Materials

The following is available online at https://www.mdpi.com/article/10.3390/foods10123098/s1, Table S1: Survey on sociodemographic profile, health status, knowledge and perception of sprouts in adult population from Northwestern Mexico.
