# Supplementary material for: Consumption of Sprouts and Perceptions of Their Health Properties in a Region of Northwestern Mexico
Source: Foods. 2021 Dec 14;10(12):3098. doi: 10.3390/foods10123098 (PMC8701714; doi:10.3390/foods10123098)
Supplement: Supplementary file 1 [file foods-10-03098-s001.zip › foods-1439452-supplementary.pdf]

foods MDPI

Table S1. Survey on sociodemographic profile, health status, knowledge and perception of sprouts in adult population from Northwestern Mexico

**I. Sociodemographic profile**
1. What is your gender?<br>a. Female<br>b. Male
2. What is your age range?<br>a. 18–28<br>b. 29–39<br>c. 40–50<br>d. 51–60
3. What is your education level?<br>a. High school<br>b. University education<br>c. Postgraduate
4. What is your current occupation?<br>a. Stay at home<br>b. Full-time work<br>c. Part-time work<br>d. Student<br>e. Self-employment
5. What is your personal monthly income (MXN) range?<br>a. No salary<br>b. Less than 5,000<br>c. 5,000–15,000<br>d. 16,000–25,000<br>e. 26,000–35,000<br>f. More than 35,000
6. How many days a week are you physically active?<br>a. 5–7<br>b. 3–5<br>c. 1–3<br>d. None
7. Do you suffer, or did you suffer from any of these diseases?

a. Diabetes
b. Hypertension
c. Obesity/Overweight
d. Cancer
e. Stroke
f. Fatty liver
g. Gynaecological problems
h. Coronary heart disease
a. None of the above

8. From this list, which sprouts have you seen for sale?
   a. Broccoli
   b. Alfalfa
   c. Soy
   d. Amaranth
   e. Chia
   f. Radish
   g. Mung bean
   h. Clover
   i. None

9. From this list, which sprouts have you consumed?
   a. Broccoli
   b. Alfalfa
   c. Soy
   d. Amaranth
   e. Chia
   f. Radish
   g. Mung bean
   h. Clover
   i. None

10. On a five-point Likert scale, what do you think of the following characteristics of sprouts?
   "1": I like it very much, "2": I like it, "3": It is indifferent to me, "4": I dislike it, "5": I dislike it very much
   a. Flavour
   b. Texture
   c. Odour
   d. Colour
   e. Consistency

11. How often do you consume sprouts?
   a. 4–7 times a week

b. 1–3 times a week
c. Once a month
d. Once every six months
e. Once a year
f. Never

12. To what would you attribute the beneficial effect of sprouts?
a. Fibre
b. Minerals
c. Vitamins
d. Polyphenols
e. All of the above
f. I don´t know

13. Regular consumption of sprouts is linked to a lower likelihood of developing diseases such as diabetes, hypertension and cancer. Did you know this?
a. Yes
b. No

14. Regarding the previous question, would you consume or increase your consumption of sprouts to prevent these diseases?
a. Yes
b. No
